# DgeaHeatmap: an R package for transcriptomic analysis and heatmap generation

**DOI:** 10.1093/bioadv/vbaf194

**Published:** 2025-08-20

**Authors:** Leonie J Lancelle, Phani S Potru, Björn Spittau, Susanne Wiemann

**Affiliations:** Department of Anatomy and Cell Biology, Medical School OWL, Bielefeld University, Bielefeld 33615, Germany; Department of Anatomy and Cell Biology, Medical School OWL, Bielefeld University, Bielefeld 33615, Germany; Department of Anatomy and Cell Biology, Medical School OWL, Bielefeld University, Bielefeld 33615, Germany; Department of Anatomy and Cell Biology, Medical School OWL, Bielefeld University, Bielefeld 33615, Germany

## Abstract

**Motivation:**

The growing use of transcriptomic data from platforms like Nanostring GeoMx DSP demands accessible and flexible tools for differential gene expression analysis and heatmap generation. Current web-based tools often lack transparency, modifiability, and independence from external servers creating barriers for researchers seeking customizable workflows, as well as data privacy and security. Additionally, tools that can be utilized by individuals with minimal bioinformatics expertise provide an inclusive solution, empowering a broader range of users to analyze complex data effectively.

**Results:**

Here, we introduce Differential Gene Expression Analysis and Heatmaps (DgeaHeatmap), an R package offering streamlined and user-friendly functions for transcriptomic data analysis particularly yielded by Nanostring GeoMx DSP instruments. The package supports both normalized and raw count data, providing tools to preprocess, filter, and annotate datasets. DgeaHeatmap leverages Z-score scaling and k-means clustering for customizable heatmap generation and incorporates a workflow adapted from GeoMxTools for handling raw Nanostring GeoMx DSP data. By enabling server-independent analyses, the package enhances flexibility, transparency, and reproducibility in transcriptomic research.

**Availability and implementation:**

The package DgeaHeatmap is freely available on GitLab (https://gitlab.ub.uni-bielefeld.de/spittaulab/Dgea_Heatmap_Package.git).

## 1 Introduction

Differential Gene Expression Analysis and Heatmaps (DgeaHeatmap) is an R package specifically designed to simplify the analysis of GeoMx DSP data as well as other types of transcriptomic data, offering functions for differential gene expression analysis and heatmap generation. Methods to analyze transcriptomic data typically produce count data, which require preprocessing and normalization to enable meaningful comparisons. The data used for demonstration purposes in this paper were obtained from previously published datasets generated through GeoMx™ Digital Spatial Profiling (DSP) to investigate spatial gene expression (Reeves *et al.* 2021, Griswold *et al.* 2025). This powerful technique combines high-resolution spatial profiling with quantitative transcriptomic analysis, allowing researchers to measure gene expression in specific regions of interest within tissue samples. The resulting data often require specialized tools to preprocess, filter, and visualize. Additionally, we also provide evidence that DgeaHeatmap can be adapted to analyze datasets beyond GeoMx™ Digital Spatial Profiling (S1).

DgeaHeatmap was developed to address these requirements by integrating methods to process raw count data from the GeoMx™ platform and generate publication-ready heatmaps. It adapts workflows from the established “Analyzing GeoMx-NGS RNA Expression Data with GeomxTools” by Reeves *et al.* (2021) and standR for handling raw Nanostring GeoMx DSP data, while also supporting normalized datasets. While the R packages, GeomxTools and standR provide functions for processing of GeoMx-NGS data, more user-friendly functions are needed to simplify the process as well as functions needed for downstream analyses and visualization of the data (Reeves *et al.* 2021, Liu *et al.* 2024). DgeaHeatmap provides the necessary tools for Z-score scaling, k-means clustering, and customizable annotation, ensuring that researchers can perform spatial gene expression and transcriptomic analyses easily and effectively.

Unlike web-based tools, DgeaHeatmap is server-independent, providing enhanced transparency, adaptability, and flexibility for users. By including functions for both normalized and raw count data workflows, this package simplifies the end-to-end analysis, making it accessible even to researchers with limited R programming expertise.

## 2 System and methods

DgeaHeatmap is an R package developed in and for usage in RStudio using R (Posit Team 2024) (version 2024.4.0.735), with the purpose to gain easy-to-use functions for heatmap generation and differential gene expression analysis. While the functions to create heatmaps are based on Z-Score scaling and k-means clustering, the functions for analyzing raw Nanostring GeoMx DSP data are adapted from the workflow established by Reeves *et al.* in 2021. Furthermore, this package includes functions to simplify the analysis and to extract the expression data and annotation data from the raw data. A subsequent differential gene expression analysis can optionally be performed using limma voom, DESeq2, or edgeR (Law *et al.* 2014, Love *et al.* 2014, Ritchie *et al.* 2015, Chen *et al.* 2025). DgeaHeatmap was developed to streamline the analysis of transcriptomic data, enabling users to perform these tasks efficiently and independently. One of the major advantages is that R and the package are independent of servers, unlike currently available webtools that serve a similar purpose. Additionally, the process of data analysis using DgeaHeatmap is much more transparent and easily modifiable.

The package includes functions to help both with the analysis of normalized read counts, as well as functions to simplify the extraction of counts data generated through Nanostring GeoMx DSP and other transcriptomic approaches.

## 3 Algorithm

The algorithm is composed of two parts, the first being the import and preparation of normalized read counts, raw Nanostring GeoMx DSP data or raw counts, and the second part aimed at building heatmaps based on the prepared data. Every file that can be read into R can be used as input if it contains gene names, sample names and raw or normalized counts depending on the analysis. An example for usage of the DgeaHeatmap package independent of GeoMx DSP can be found in the Supplement Material (S1), available as supplementary data at *Bioinformatics Advances* online, conducted with previously published micro-array data available as accession number GSE115652 on NCBI GEO (Zöller *et al.* 2018).

The first part of the algorithm is subdivided into functions to support the import and data preprocessing. To generate heatmaps, normalized data is suggested as input, these can be read into R for example as CSV files. Next, the dataframe is converted into a matrix before parameters are chosen in order to possibly extract specific sample groups from the original matrix or to filter and pre-process the data. Optionally, an individual matrix containing only certain sample groups can be created, before the actual functions for preprocessing and filtering are used. If Nanostring GeoMx DSP data is available, the DCC, PKC, and XLSX files can be loaded into RStudio to generate an object of the class “GeoMxSet Object,” preprocessed and filtered, before extracting the counts matrix and annotation data. Next, the sample numbers in the count matrix are exchanged with their corresponding sample names for better clarification and allocability. Subsequently, a differential gene expression analysis can be conducted utilizing functions based on limma voom, DESeq2, or edgeR (Law *et al.* 2014, Love *et al.* 2014, Ritchie *et al.* 2015, Chen *et al.* 2025). Furthermore, the differential expression analysis may also be conducted using data generated through other experiments. However, the data has to be raw counts for the normalization factors needed for the differential expression analysis to be calculated.

The second part of the algorithm starts out by visualizing the data distribution of the preprocessed data as a histogram to check for normal distribution. An elbow plot is generated, plotting the variation as a function of the number of clusters, to show the ratio of between-group variance to total variance. The elbow of the plot is then chosen as the number of clusters in the subsequently generated heatmaps in the last step.

## 4 Implementation

**Figure 1.**
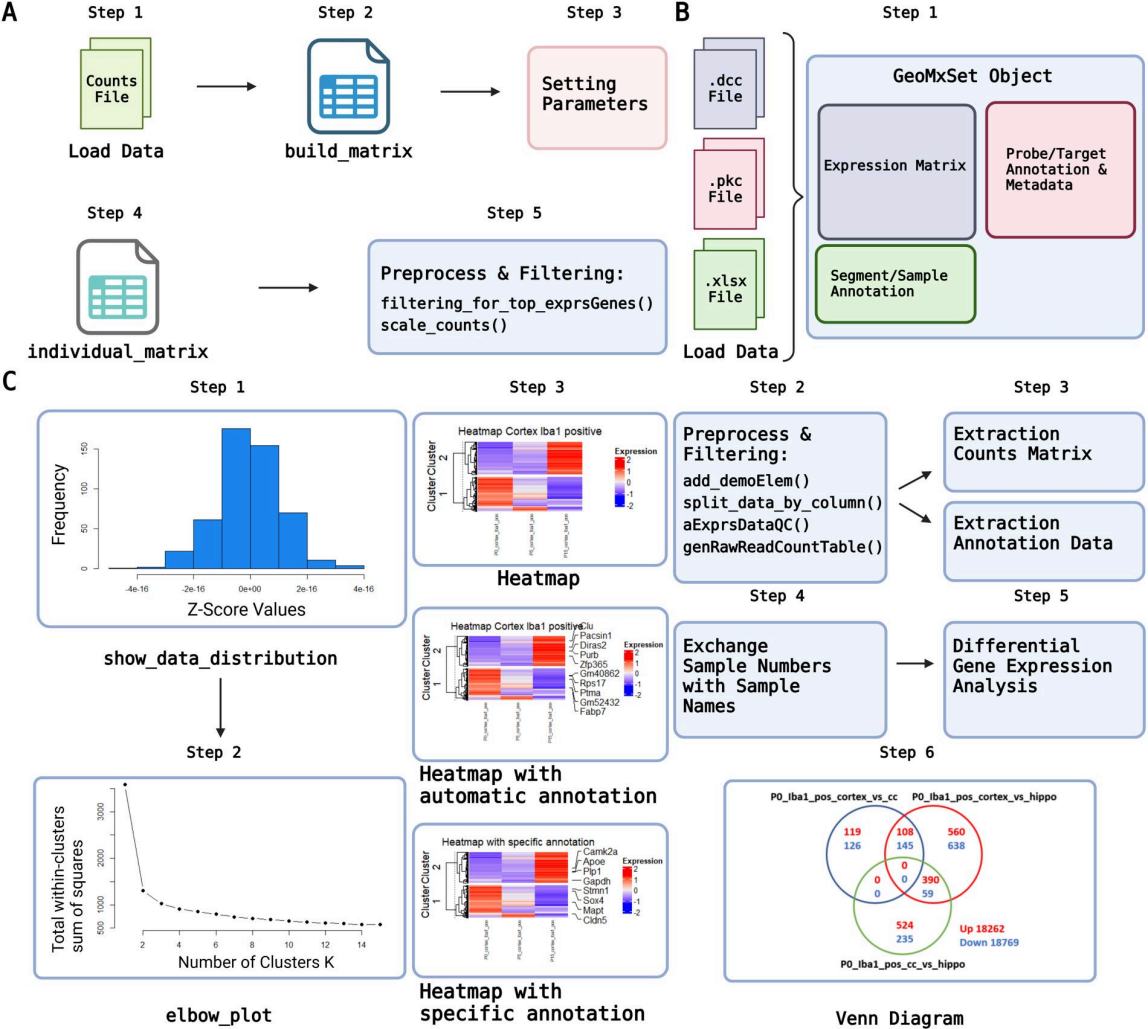
Step-by-step workflow of a data analysis using either Read Counts or raw Nanostring GeoMx DSP data files. (A) Description of the steps starting with Read Counts using functions from the package DgeaHeatmap. (B) The raw files generated through Nanostring GeoMx DSP are loaded, preprocessed, and filtered in order to then extract the Read Counts of all evaluated genes and to perform a differential gene expression analysis. (C) Using prepared data, the depicted functions can be utilized to visualize the data distribution and to choose the fitting number of clusters for the dataset. Heatmaps can be generated showing no annotation, automatically generated annotation, or specifically chosen annotation as desired. Created with BioRender (www.biorender.com).

The package of DgeaHeatmap includes all necessary steps from data import to the final output results. Figure 1 depicts this workflow. In step 1 of A, a counts file is loaded into R, which is then converted into a matrix during step 2, by utilizing the function “build_matrix.” In step 3, users can set parameters for the subsequent analysis. These can include, e.g. a parameter representing the top number of most variable genes, as well as parameters to select only specified samples from the original dataset. On this basis, step 4 enables the generation of a matrix containing only certain columns, that were extracted from the original matrix using the function “individual_matrix” and a previously set parameter. In step 5, the data is further preprocessed and filtered by the functions “filtering_for_top_exprsGenes” and “scale_counts,” in which the former extracts the top n most variably expressed genes across the samples, while the later enables an easy Z-count scaling of the counts within the matrix respectively.

If the analysis is conducted using raw Nanostring GeoMx DSP data, the workflow starts out at B by reading in the DCC files, a PKC file, as well as an XLSX file, containing the annotation information of the samples. Adapting a workflow published by Nanostring, the files are read into RStudio and summarized into one object of class “GeoMxSet Object,” which then contains an expression matrix, segment/sample annotation, probe/target annotation and metadata (Reeves *et al.* 2021). In step 2, the data is preprocessed and filtered using the functions “add_demoElem,” “splits_data_by_column,” “aExprsDataQC,” and “genRawReadCountsTable.” Subsequently, both the counts matrix and the annotation data are extracted from the “GeoMxSet Object,” for this e.g. the function “genRawReadCountsTable” can be utilized. In step 4 of the workflow, the sample numbers in the count matrix are exchanged with their corresponding sample names. Finally, in step 5, a differential gene expression analysis is conducted using either “DGEALimma,” “DGEADESeq2,” or “DGEAedgeR.” The results can then be extracted using “extractDEGenes” and “summarize_edgeR_DEA,” before visualizing the results for example as a Venn diagram in step 6.

In both cases of previous data processing, the results can subsequently be plotted as heatmaps, as depicted in **C**. For this, the data distribution can first be visualized using the function “show_data_distribution” to generate a histogram. In step 2, the amount of clusters k is determined for the k-means clustering through generating an elbow plot, from which the “elbow” is chosen as number of clusters k. In the last step, three different heatmaps can be created, which differ especially in their annotation. The functions “print_heatmap,” “function_complexHeatmap_var,” and “adv_heatmap” allow the user to create heatmaps without annotation, with automatically generated annotation, or with specific annotation. By calculating the variances in the expression of each gene across the samples, a user-defined number of genes with the highest variance in their respective cluster are used for the automatic annotation of the heatmap.

**Figure 2.**
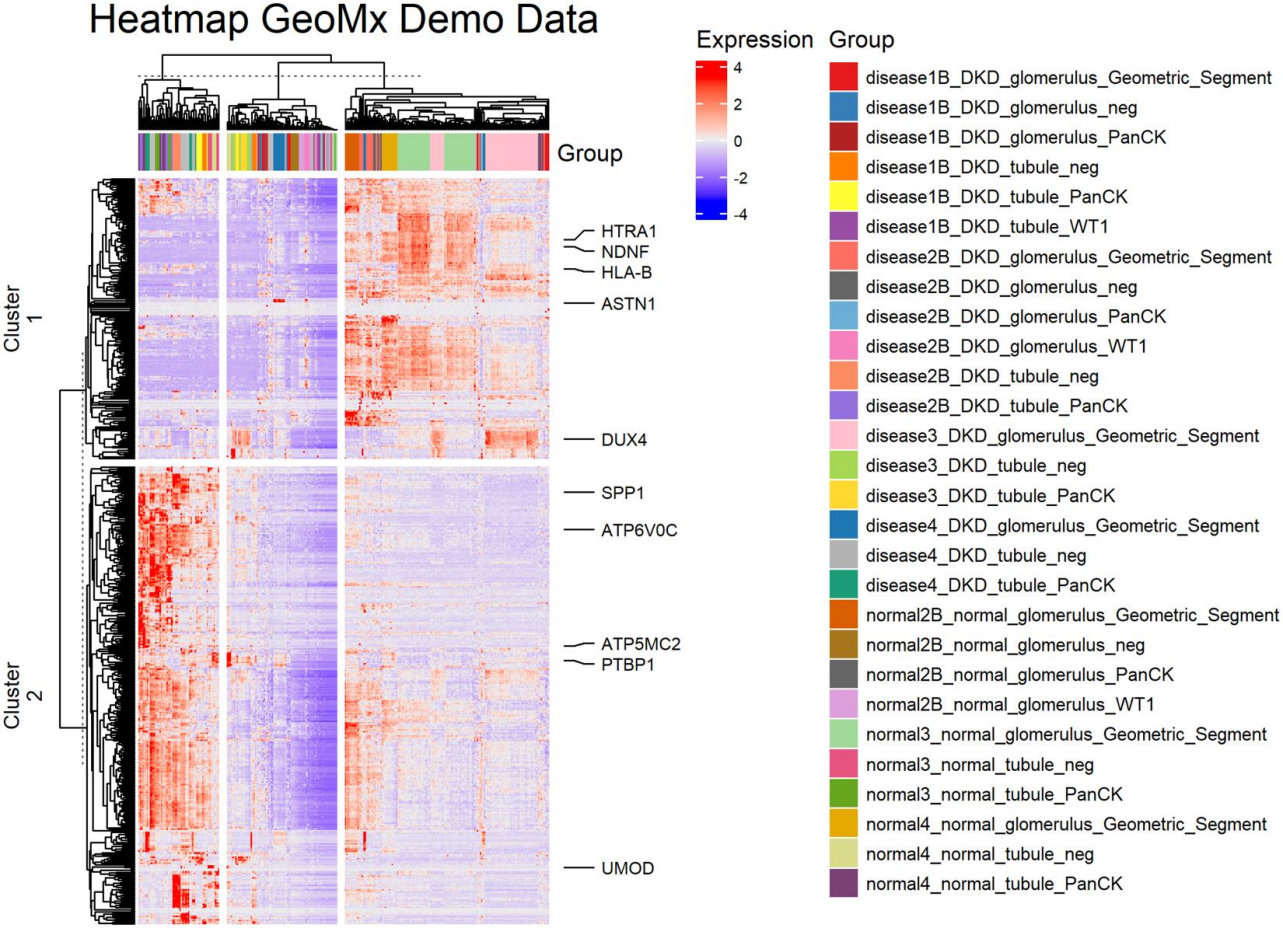
Heatmap of the entire GeoMx kidney demonstration dataset pre-processed, normalized and analyzed using DgeaHeatmap in RStudio with R (Posit Team 2024) (version 2024.4.0.735). The heatmap illustrates the 500 most variably expressed genes of the dataset, divided into two clusters with 10 annotations of the most variable genes per cluster. The seed for clustering was defined as seed = 1. The color palette was defined through colorPalette = “RdBu.”

In this example GeoMx-NGS mRNA expression data prepared by Nanostring was used for demonstration. The original data contained a DCC, a PKC, and an XLSX file created with the human whole transcriptome atlas (WTA) assay. These files contained information about the expressed count data, sequencing quality metadata, probe assay metadata, and annotation information of four diabetic kidney disease and three healthy kidney tissue samples. The regions of interest (ROI) were set up to focus on either the tubules or glomeruli of the kidney tissue and were further segmented into distal and proximal tubule areas of illumination (AOI) (Reeves *et al.* 2021). Heatmaps generated from this dataset could depict either the entire dataset or focus on comparing only specific samples (Fig. 2). To get a much more customized heatmap, the function “adv_heatmap” can be used. This enables the generation of heatmaps based on k-means clustering and hierarchical clustering, optional clustering of rows and columns, available and customizable column annotation, as well as optional changes of all the sizes of the graphics and the font.

The heatmap depicted in Fig. 2 shows an example of the entire demo dataset of Nanostring GeoMx. All analyzed kidney tissue samples were grouped by ROI and AOI for greater clarity.

The source code of the DgeaHeatmap package is available to users on https://gitlab.ub.uni-bielefeld.de/spittaulab/Dgea_Heatmap_Package.git, where all performed unit tests can also be inspected. The package is currently in preparation for submission to Bioconductor.

## 5 Discussion

The presented R package, DgeaHeatmap, provides a user-friendly and elegant approach to deal with GeoMx DSP raw data and to generate heatmaps. One of the main benefits of this package is its independence from the Nanostring web-tool and its tailored approach for handling GeoMx DSP data. The package provides an easy-to-use approach to extract raw read counts from DCC, PKC, and annotation files, to allow a more flexible and adaptable analysis outside of the Nanostring platform. Additionally, it can also be used for analysis of other kinds of transcriptomic data, as shown (S1). Furthermore, DgeaHeatmap combines simple functions for fast and easy heatmap generation. The package includes functions for automatic annotation and easy customization, while requiring less knowledge of R, thus being more intuitive to use for less bioinformatically advanced scientists.

Overall, DgeaHeatmap provides a solution for quick and server-independent analyses of transcriptomic data, while being much more transparent and easier to modify. Since the package is based on R it can be easily combined with other R pipelines and tools. It can further be run locally, enhancing data privacy and security while enabling the analysis of highly sensitive personal data.

In the future, the determination of the number of clusters k could be further simplified and automated. For this purpose, a function could be added to choose the optimal number of clusters through generating a cluster index for example, by using the Krzanowski-Lai index (Krzanowski and Lai 1988).

## Supplementary Material

vbaf194_Supplementary_Data

## Data Availability

The transcriptome data used are publicly available and obtainable through the R package GeoMxWorkflows (Griswold *et al.* 2025). The R package DgeaHeatmap is freely available on GitLab: https://gitlab.ub.uni-bielefeld.de/spittaulab/Dgea_Heatmap_Package.git
