# Supplementary material for: DgeaHeatmap: an R package for transcriptomic analysis and heatmap generation
Source: Bioinform Adv. 2025 Aug 20;5(1):vbaf194. doi: 10.1093/bioadv/vbaf194 (PMC12401572; doi:10.1093/bioadv/vbaf194)
Supplement: vbaf194_Supplementary_Data [file vbaf194_supplementary_data.zip › Supplementary Material_Lancelle 2025.pdf]

SupplementaryExampleDgeaHeatmapPaper

Leonie Lancelle

2025-07-14

Read in of data as CSV file:

```
# Read in of data as csv file for example like this:
# raw_counts <- read.csv(ArrayCounts.csv)

# use build_matrix() to build a matrix from the counts
raw_matrix <- build_matrix(raw_counts, 1)

# Replace "." in the colnames and rownames with "_" to avoid errors and missfunction of Code
colnames(raw_matrix) <- gsub("\\.", "_", colnames(raw_matrix))
rownames(raw_matrix) <- gsub("\\.", "_", rownames(raw_matrix))
```

Generate a Metadata File from the Counts File

```
# Set group names as list
groups <- c("Cntrl", "Tgfb1_24h")

# Get Count Datas Sample Names
sample_names <- c(colnames(raw_matrix))

# Match each sample name to the correct group name
group_assignment <- sapply(sample_names, function(sample) {
  matched <- groups[sapply(groups, function(g) grepl(g, sample))]
  if (length(matched) > 0) matched[1] else NA
})

# Create Metadata File as dataframe
sample_metadata <- data.frame(Group = group_assignment, row.names = sample_names)

# Confirmation that colnames of the Counts match rownames of the Metadata File
all(colnames(raw_matrix) == rownames(sample_metadata))
```

```
## [1] TRUE
```

Check ups before Differential Expression Analysis:

```r
#check if all column names of data are in rownames of metadata
all(colnames(raw_matrix) %in% rownames(sample_metadata))
```

```
## [1] TRUE
```

```r
#check if the order of the data column names == order of metadata rownames
all(colnames(raw_matrix) == rownames(sample_metadata))
```

```
## [1] TRUE
```

DEA with Limma

```r
sample_metadata <- as.data.frame((sample_metadata))

comparisons <- list(
  "cntrl_vs_Tgfb1_24h" = c("Cntrl", "Tgfb1_24h"),
  "Tgfbeta1_24h_vs_cntrl" = c("Tgfb1_24h", "Cntrl")
  )

groupingColumns <- c("Group")
DGEA_resultsLimma <- DGEALimma(raw_matrix, sample_metadata, grouping_columns = groupingColumns)

results_all_DEG <- decideTests(DGEA_resultsLimma$fit)

summary(results_all_DEG)
```

```
##        Cntrl - Tgfb1_24h
## Down                 608
## NotSig             22026
## Up                   666
```

```r
result1 = topTable(DGEA_resultsLimma$fit, coef= "Cntrl - Tgfb1_24h", number = Inf,
                   adjust.method = "fdr") %>%
  as.data.frame() # differentially expressed genes are obtained by topTreat() function
```

Filter results to get significantly differentially expressed genes:

```r
topUpLimma <- result1[which(result1$logFC > 0),] [1:50,] # up reg top 100
```

```r
topDownLimma <- result1[which(result1$logFC < 0),] [1:50,] # down reg top 100
```

The significantly expressed genes can be extracted like this:

```r
resultSig <- result1 %>%
  dplyr::mutate(isSignificant = case_when(
    adj.P.Val < 0.05& abs(logFC) >1 ~TRUE,
    TRUE ~ FALSE # If condictions in the line above are not met, gene is not DE.
  ))

sigDEresults <- resultSig %>%
  dplyr::filter(isSignificant == TRUE)
```

```r
venn.plot <- vennDiagram(results_all_DEG,
imagetype = "tiff",
include=c("up", "down"), mar=rep(1,4), cex=c(1,1,0.7), lwd=1,
counts.col=c("red", "blue"),
circle.col = c("red", "blue", "green3"))
```

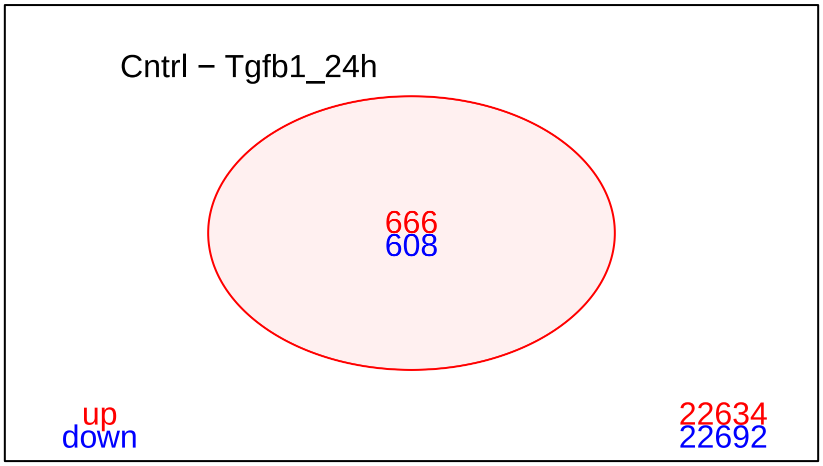

Perform Differential Expression Analysis based on DESeq2:

```r
comparisons <- list(
  "cntrl_vs_Tgfb1_24h" = c("Cntrl", "Tgfb1_24h")
)
int_counts <- round(raw_matrix) # Counts have to be integer for DEseq2
groupingColumns <- c("Group")
results_DESeq2 <- DGEA_DESeq2(int_counts, sample_metadata, groupingColumns, comparisons = comparisons)
```

```
## converting counts to integer mode

## estimating size factors

## estimating dispersions

## gene-wise dispersion estimates
```

```
## mean-dispersion relationship

## final dispersion estimates

## fitting model and testing
```

```
results_list_d <- results_DESeq2$results
```

The results are then further extraced as in the following examples:

```
up_genes <- extractDEGenes(results_list_d, contrasts, only_up = TRUE)
down_genes <- extractDEGenes(results_list_d, contrasts, only_down = TRUE)
sig_genes <- extractDEGenes(results_list_d, contrasts, only_sig = TRUE)
state_mat <- extractDEGenes(results_list_d, contrasts, up_down = TRUE)
```

The results can then be depicted as Venn Diagrams or used for further analysis.

```
state_mat <- data.frame(state_mat)
venn.plot <- vennDiagram(state_mat,
                         imagetype = "tiff",
                         include=c("up", "down"), mar=rep(1,4), cex=c(1.5,1,0.7), lwd=1,
                         counts.col=c("red", "blue"),
                         circle.col = c("red", "blue", "green3"))
```

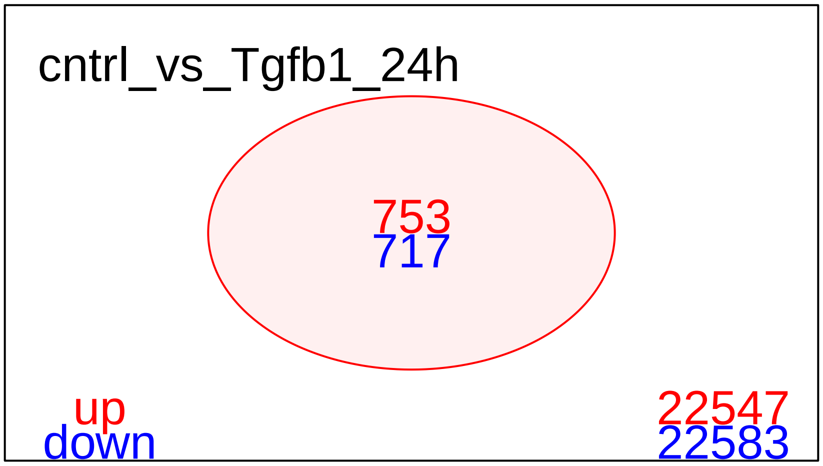

The significantly expressed genes can be extracted like this:

```r
results_DESeq2 <- results_list_d$cntrl_vs_Tgfb1_24h
resultSigDESeq2 <- results_DESeq2 %>%
  dplyr::mutate(isSignificant = case_when(
    padj < 0.05& abs(log2FoldChange) >1 ~TRUE,
    TRUE ~ FALSE # If condictions in the line above are not met, gene is not DE.
  ))

sigDEresultsDESeq2 <- resultSigDESeq2 %>%
  dplyr::filter(isSignificant == TRUE)
```

Filter results to get significantly differentially expressed genes:

```r
topUpDESeq2 <- results_DESeq2[which(results_DESeq2$log2FoldChange > 0),] [1:50,] # up reg top 100
```

```r
topDownDESeq2 <- results_DESeq2[which(results_DESeq2$log2FoldChange < 0),] [1:50,] # down reg top 100
```

Perform Differential Expression Analysis based on edgeR:

```r
comparisons <- list(
  "cntrl_vs_Tgfb1_24h" = c("Cntrl", "Tgfb1_24h")
)
groupingColumns <- c("Group")
results_edgeR <- DGEAedgeR(int_counts, sample_metadata, groupingColumns, comparisons, prefix = "DEA")

edgeR_summary <- summarize_edgeR_DEA(results_edgeR)
```

Extract the output from edgeR:

```r
results_edgeR <- results_edgeR$results$cntrl_vs_Tgfb1_24h
resultSigedgeR <- results_edgeR %>%
  dplyr::mutate(isSignificant = case_when(
    FDR < 0.05& abs(logFC) >1 ~TRUE,
    TRUE ~ FALSE # If condictions in the line above are not met, gene is not DE.
  ))

sigDEresultsedgeR <- resultSigedgeR %>%
  dplyr::filter(isSignificant == TRUE)
state_matrix <- edgeR_summary$classified_results %>%
  lapply(function(df) df$decision) %>%       # extract the -1/0/+1 vector for each contrast
  do.call(cbind, .)                          # bind into a matrix
colnames(state_matrix) <- names(edgeR_summary$classified_results)
```

The results can then be depicted as Venn Diagrams or used for further analysis.

```r
state_matrix <- edgeR_summary$classified_results %>%
  lapply(function(df) df$decision) %>%       # extract the -1/0/+1 vector for each contrast
  do.call(cbind, .)                          # bind into a matrix
colnames(state_matrix) <- names(edgeR_summary$classified_results)
venn.plot <- vennDiagram(state_matrix,
                         imagetype = "tiff",
```

```
                                include=c("up", "down"), mar=rep(1,4), cex=c(1.5,1,0.7), lwd=1,
                                counts.col=c("red", "blue"),
                                circle.col = c("red", "blue", "green3"))
```

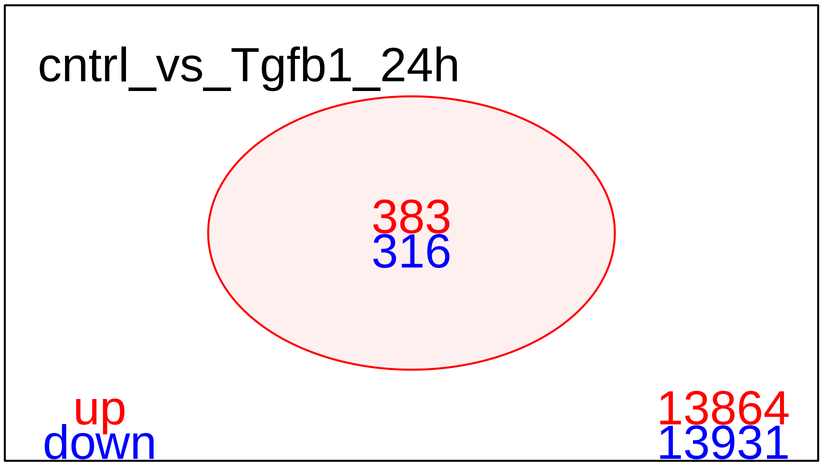

Filter results to get significantly differentially expressed genes:

```
topUpedgeR <- results_edgeR[which(results_edgeR$logFC > 0),] [1:50,] # up reg top 100
```

```
topDownedgeR <- results_edgeR[which(results_edgeR$logFC < 0),] [1:50,] # down reg top 100
```

After the differential Expression Analysis, the results can be visualized in a heatmap using the functions from DgeaHeatmap. First the normalized counts have to be extraced from the results of the Differential Expression Analysis.

```
y <- DGEA_resultsLimma$normFactors
CPM <- edgeR::cpm(y)
df_normCounts <-data.frame("genes" = row.names(CPM), CPM)
```

The next steps can vary based what is wished to be archieved. Here are some examples using the micro-array data.

- differentially expressed genes are extracted and depicted in a heatmap

```r
# Generate List of differentially expressed genes
includeList <- c(topUpLimma$ID, topDownLimma$ID)
# Exctract matrix only with differentially expressed genes
extrMatrix <- subset(df_normCounts, rownames(df_normCounts) %in% includeList)
# delete duplicate gene name column
extrMatrix <- as.matrix(extrMatrix[,-1])
```

- next the data has to be scaled:

```r
scaled_counts <- scale_counts(extrMatrix)
```

- show data distribution:

```r
show_data_distribution(scaled_counts)
```

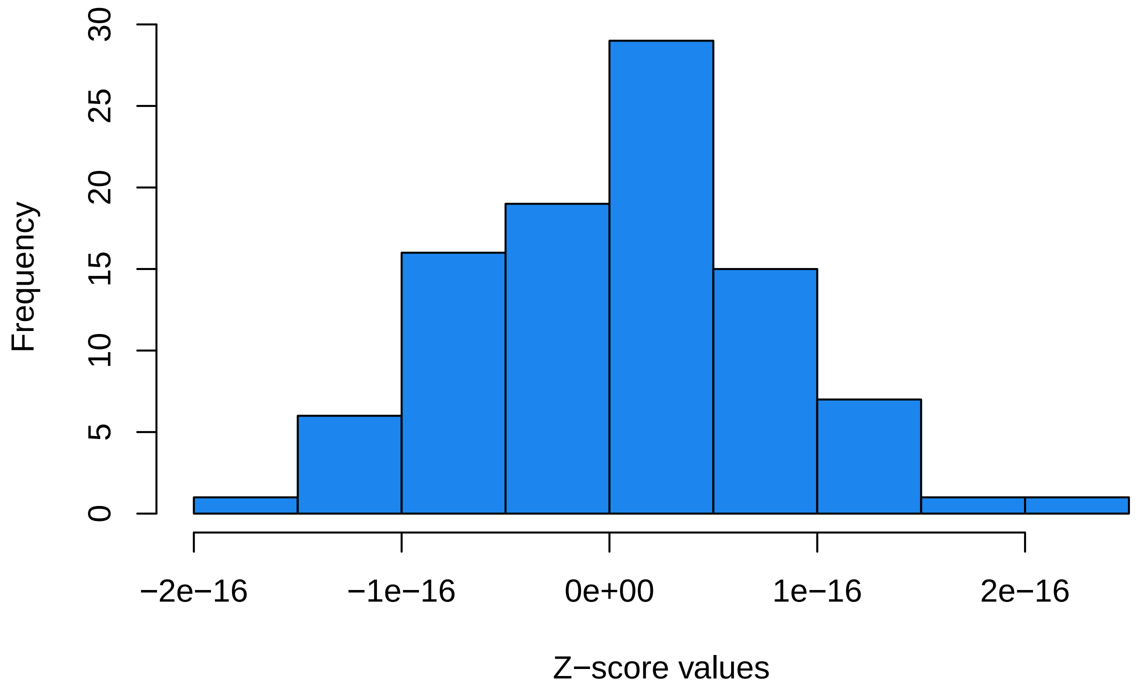

- generate elbow Plot for Gene Clustering:

```r
seed <- 1 # setting a seed for a reproducible outcome
elbow_plot(seed, scaled_counts)
```

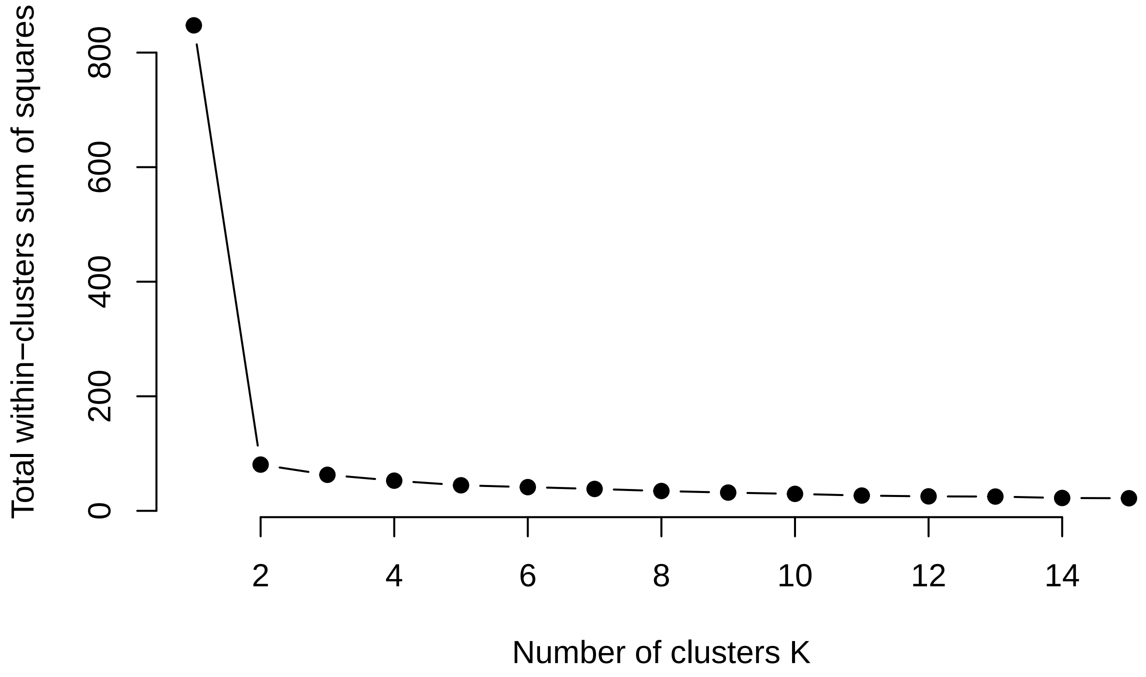

Genes will therefore be clustered in 2 clusters if k-means clustering is chosen.

- generate elbow Plot for Sample Clustering:

```
maxK <- 4                                  # only 4 samples therefore a max of 4 clusters possible
seed <- 1                                  # setting a seed for a reproducible outcome
transposed_matrix <- t(scaled_counts)      # transposes matrix
elbow_plot(seed, transposed_matrix, maxK)
```

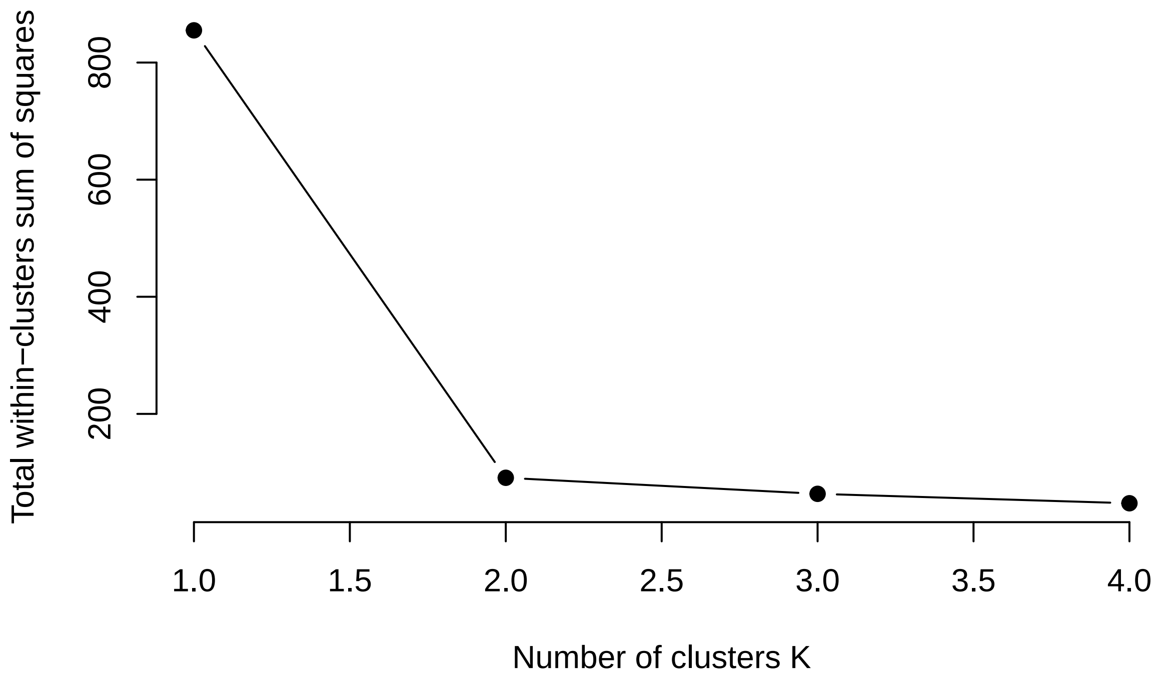

Samples will also be clustered into 2 clusters if k-means clustering is chosen for the heatmap.

```
# set a list with the Groups and choose colors for them
group_colors <- list(Group = c("Cntrl" = "#E78AC3", "Tgfb1_24h" = "#6b6ecf"))
row_anno_list <- list("Atp8b4", "Ptpn22", "Ccl3", "Lilrb3", "Pdxk",
                      "Tmem119", "Cx3cr1", "Olfml3", "Apoc1", "Mrc1")
```

- and advanced heatmap can be created based on K-means clustering

```
# parameters and their options in adv_Heatmap()
ncounts_matrix <- scaled_all_counts # input matrix
seed <- 1                          # sets seed
column_name <- "Heatmap K-means Clustered"# name for heatmap
colorPalette <- "RdBu"             # available color palettes from RColorBrewer ()
cluster_method <- "kmeans"         # cluster methods, either "hierarchical" or "kmeans"
distance_method <- "euclidean"     # distance matrix method
cluster_rows <- TRUE               # clustering of rows
cluster_columns <- FALSE           # clustering of columns
k_row = 3                          # splitting of rows in heatmaps using k-means
k_col = NULL                       # splitting of columns in heatmaps using k-means
sample_metadata <- newMetaData     # metadata information
annotation_colors <- group_colors  # list containing group annotation info
annotation_name_side = "right"     # side of annotation name
show_row_names <- FALSE            # show of rownames
show_column_names = TRUE           # show of column names
row_annotation = TRUE              # row annotation
```

```r
row_annotation_method = "specific" # set row_annotation method
row_anno_names = row_anno_list     # list of specific genes for the row annotation
row_anno_number = 4                # number of automatic row annotations per cluster
fontsize_title = 15                # fontsize the heatmap title
fontsize_rowAnnotation = 8         # row annotation
fontsize_columnNames = 8           # column names
fontsize_rowNames = 4              # row names,
fontsize_cluster_labels = 8        # cluster labels
fontsize_group_annotation = 9      # group annotation title
fontsize_group_annotation_legend = 9 # group annotation legend title
fontsize_group_annotation_labels = 8 # annotation legend labels
fontsize_heatmap_legend = 9        # heatmap legend
fontsize_heatmap_legend_labels = 8 # heatmap legend labels
title_heatmapLegend = "Expression" # changeable title of the legend, default "Expression"
WidthNum = 6                       # heatmap width
HeightNum = 8                      # heatmap height
UnitSize = "cm"                    # heatmap unit for sizes

hm <- adv_Heatmap(ncounts_matrix, seed = seed, column_name = column_name, colorPalette = colorPalette,
                  cluster_method = cluster_method, cluster_rows = cluster_rows,
                  cluster_columns = cluster_columns, k_row = k_row, k_col = k_col,
                  sample_metadata = sample_metadata, annotation_colors = annotation_colors,
                  annotation_name_side = annotation_name_side, show_row_names = show_row_names,
                  show_column_names = show_column_names, row_annotation = row_annotation,
                  row_annotation_method = row_annotation_method, row_anno_names = row_anno_names,
                  row_anno_number = row_anno_number, fontsize_title = fontsize_title,
                  fontsize_rowAnnotation = fontsize_rowAnnotation,
                  fontsize_columnNames = fontsize_columnNames, fontsize_rowNames = fontsize_rowNames,
                  fontsize_cluster_labels = fontsize_cluster_labels,
                  fontsize_group_annotation = fontsize_group_annotation,
                  fontsize_group_annotation_legend = fontsize_group_annotation_legend,
                  fontsize_group_annotation_labels = fontsize_group_annotation_labels,
                  fontsize_heatmap_legend = fontsize_heatmap_legend,
                  fontsize_heatmap_legend_labels = fontsize_heatmap_legend_labels,
                  title_heatmapLegend = title_heatmapLegend, WidthNum = WidthNum,
                  HeightNum = HeightNum, UnitSize = UnitSize)
```

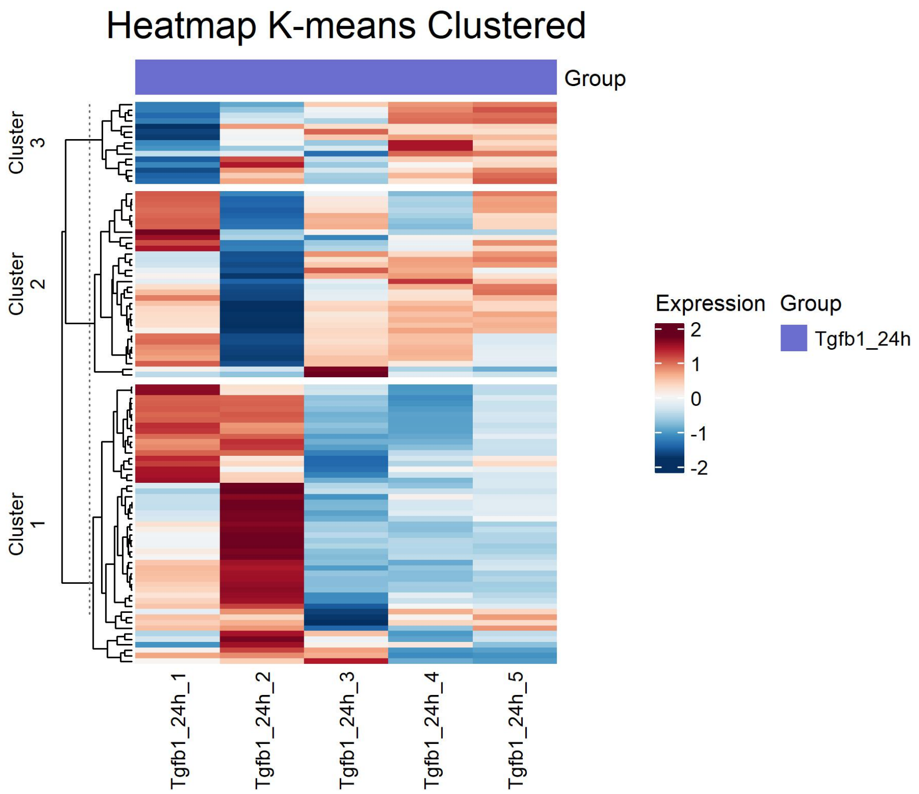

- an advanced heatmap can be generated based on hierarchical clustering

```
# parameters and their options in adv_Heatmap()
ncounts_matrix <- scaled_all_counts  # input matrix
seed <- 1                            # sets seed
column_name <- "Heatmap Hierarchically Clustered"  # name for heatmap
colorPalette <- "RdBu"               # available color palettes from RColorBrewer ()
cluster_method <- "hierarchical"     # cluster methods, either "hierarchical" or "kmeans"
```

```r
distance_method <- "euclidean"      # distance matrix method
cluster_rows <- TRUE                # clustering of rows
cluster_columns <- TRUE             # clustering of columns
k_row = NULL                        # splitting of rows in heatmaps using k-means
k_col = NULL                        # splitting of columns in heatmaps using k-means
sample_metadata <- newMetaData      # metadata information
annotation_colors <- group_colors   # list containing group annotation info
annotation_name_side = "right"      # side of annotation name
show_row_names <- FALSE             # show of rownames
show_column_names = TRUE            # show of column names
row_annotation = TRUE               # row annotation
row_annotation_method = "specific" # set row_annotation method
row_anno_names = row_anno_list      # list of specific genes for the row annotation
row_anno_number = 10                # number of automatic row annotations per cluster
fontsize_title = 15                 # fontsize the heatmap title
fontsize_rowAnnotation = 8          # fontsize of the optional row annotation
fontsize_columnNames = 8            # fontsize of the optional column names
fontsize_rowNames = 4               # fontsize of the optional row names
fontsize_cluster_labels = 8         # fontsize of the cluster labels
fontsize_group_annotation = 9       # font size of the group annotation title
fontsize_group_annotation_legend = 9 # fontsize of optional group annotation legend title
fontsize_group_annotation_labels = 8 # fontsize of optional group annotation legend labels
fontsize_heatmap_legend = 9         # fontsize of heatmap legend
fontsize_heatmap_legend_labels = 8 # fontsize of heatmap legend labels
title_heatmapLegend = "Expression" # title of the legend
WidthNum = 6                        # heatmap width
HeightNum = 8                       # heatmap height
UnitSize = "cm"                     # heatmap unit for sizes

hm <- adv_Heatmap(ncounts_matrix, seed = seed, column_name = column_name, colorPalette = colorPalette,
                  cluster_method = cluster_method, cluster_rows = cluster_rows,
                  cluster_columns = cluster_columns, k_row = k_row, k_col = k_col,
                  sample_metadata = sample_metadata, annotation_colors = annotation_colors,
                  annotation_name_side = annotation_name_side, show_row_names = show_row_names,
                  show_column_names = show_column_names, row_annotation = row_annotation,
                  row_annotation_method = row_annotation_method, row_anno_names = row_anno_names,
                  row_anno_number = row_anno_number, fontsize_title = fontsize_title,
                  fontsize_rowAnnotation = fontsize_rowAnnotation,
                  fontsize_columnNames = fontsize_columnNames, fontsize_rowNames = fontsize_rowNames,
                  fontsize_cluster_labels = fontsize_cluster_labels,
                  fontsize_group_annotation = fontsize_group_annotation,
                  fontsize_group_annotation_legend = fontsize_group_annotation_legend,
                  fontsize_group_annotation_labels = fontsize_group_annotation_labels,
                  fontsize_heatmap_legend = fontsize_heatmap_legend,
                  fontsize_heatmap_legend_labels = fontsize_heatmap_legend_labels,
                  title_heatmapLegend = title_heatmapLegend, WidthNum = WidthNum,
                  HeightNum = HeightNum, UnitSize = UnitSize)
```

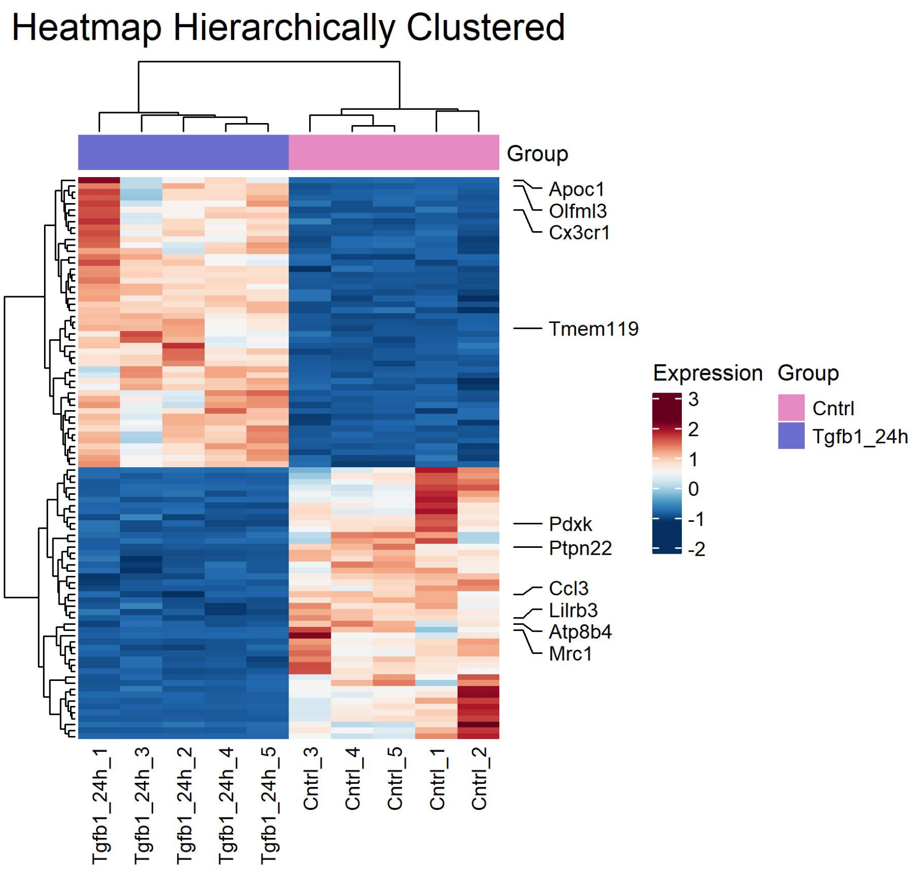

Alternatively heatmaps can be generated with all genes

```
matrixCounts <- df_normCounts
parameter1 = "Tgfb1_24h"
factors_for_individual_matrix = list(parameter1)
indiMatrix <- individual_matrix(factors_for_individual_matrix, matrixCounts)
```

- only the most variable genes could be used, for example 100 genes with highest variance

```
top_number_of_genes <- 100
varGenesMatrix <- filtering_for_top_exprGenes(indiMatrix, top_number_of_genes)
```

- next the data has to be scaled:

```
scaled_all_counts <- scale_counts(varGenesMatrix)
```

- show data distribution:

```
show_data_distribution(scaled_all_counts)
```

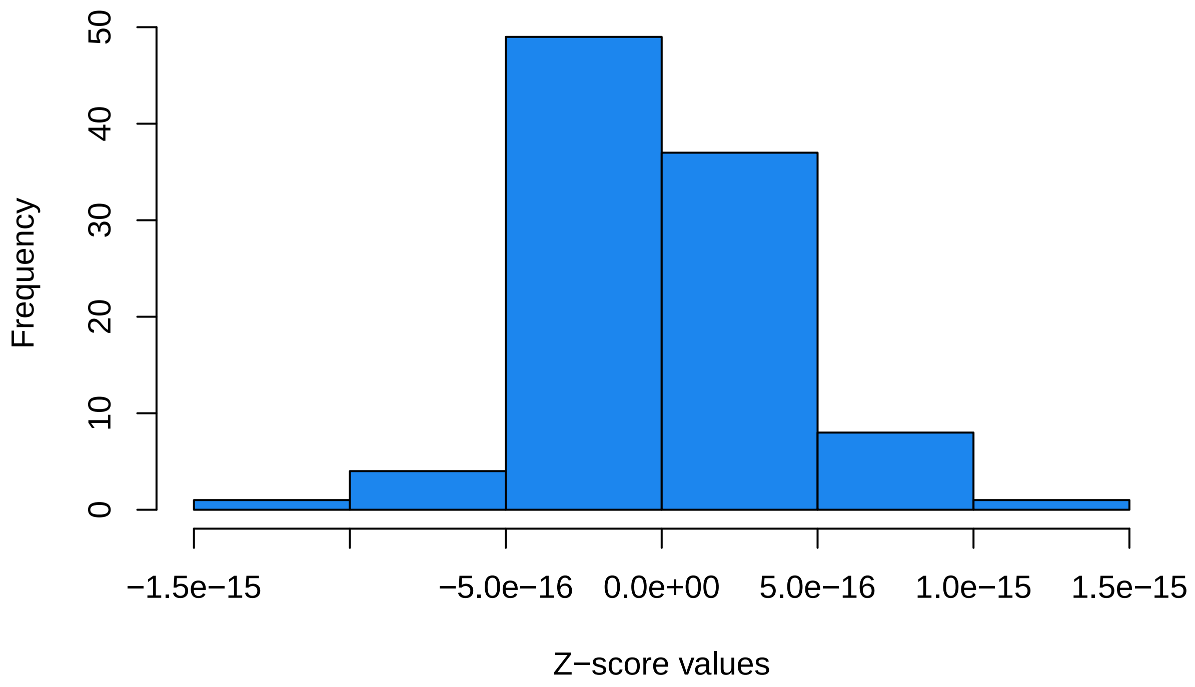

- generate elbow Plot for Gene Clustering:

```
seed <- 1 # setting a seed for a reproducible outcome
elbow_plot(seed, scaled_all_counts)
```

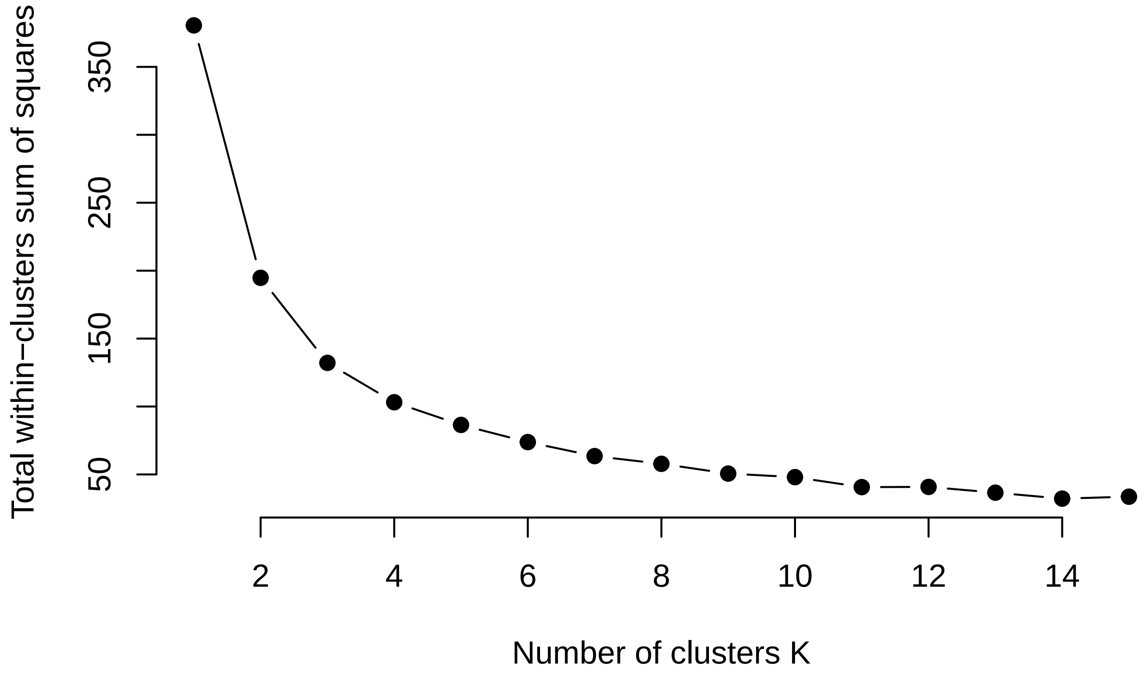

Genes will therefore be clustered in 3 clusters if k-means clustering is chosen.

- generate elbow Plot for Sample Clustering:

```
maxK <- 4                                    # only 4 samples therefore a max of 4 clusters possible
seed <- 1                                    # setting a seed for a reproducible outcome
transposed_matrix <- t(scaled_all_counts)       # transposes matrix
elbow_plot(seed, transposed_matrix, maxK)
```

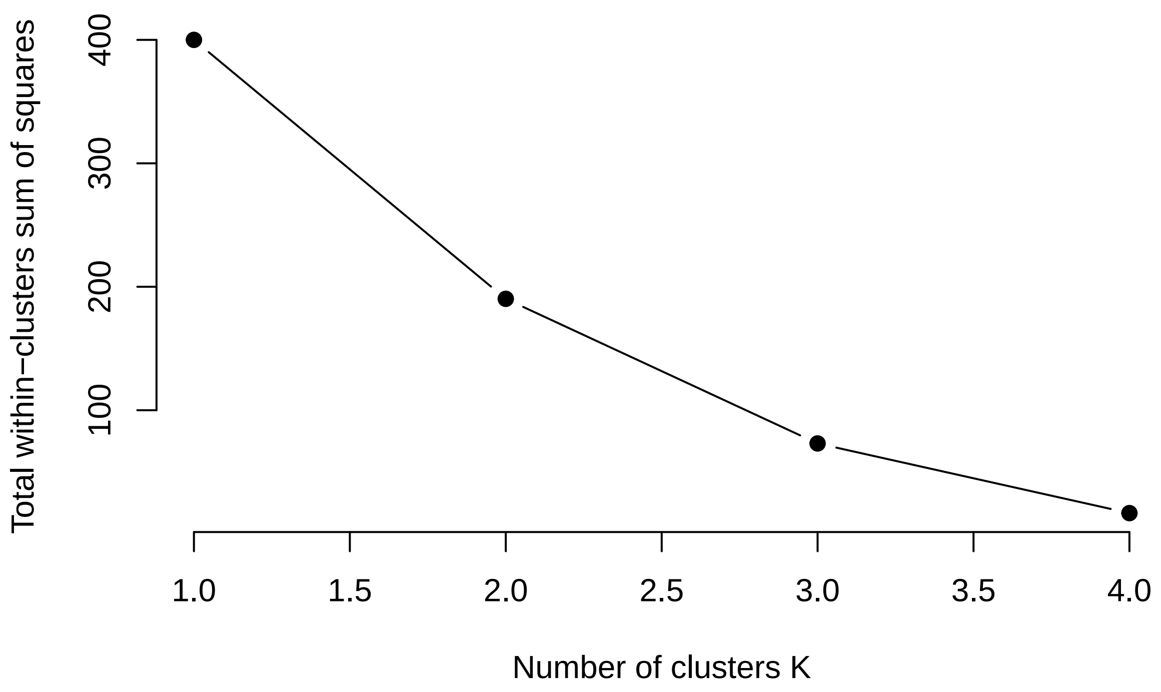

Samples will also be clustered into 0 clusters if k-means clustering is chosen for the heatmap.

```
# set a list with the Groups and choose colors for them
group_colors <- list(Group = c("Tgfb1_24h" = "#6b6ecf"))
# the meta data file has to be adjusted to only include the Tgfb1_24h samples
sample_names <- c(colnames(scaled_all_counts))

# Match each sample name to the correct group name
group_assignment <- sapply(sample_names, function(sample) {
  matched <- groups[sapply(groups, function(g) grepl(g, sample))]
  if (length(matched) > 0) matched[1] else NA
})

# Create Metadata File as dataframe
newMetaData <- data.frame(Group = group_assignment, row.names = sample_names)

# Confirmation that colnames of the Counts match rownames of the Metadata File
all(colnames(scaled_all_counts) == rownames(newMetaData))
```

```
## [1] TRUE
```

- and advanced heatmap can be created based on K-means clustering

```
# parameters and their options in adv_Heatmap()
ncounts_matrix <- scaled_all_counts # input matrix
```

```r
seed <- 1                           # sets seed
column_name <- "Heatmap K-means Clustered"# name for heatmap
colorPalette <- "RdBu"              # available color palettes from RColorBrewer ()
cluster_method <- "kmeans"          # cluster methods, either "hierarchical" or "kmeans"
distance_method <- "euclidean"      # distance matrix method
cluster_rows <- TRUE                # clustering of rows
cluster_columns <- FALSE            # clustering of columns
k_row = 3                           # splitting of rows in heatmaps using k-means
k_col = NULL                        # splitting of columns in heatmaps using k-means
sample_metadata <- newMetaData      # metadata information
annotation_colors <- group_colors   # list containing group annotation info
annotation_name_side = "right"      # side of annotation name
show_row_names <- FALSE             # show of rownames
show_column_names = TRUE            # show of column names
row_annotation = TRUE               # row annotation
row_annotation_method = "auto"      # set row_annotation method
row_anno_names = NULL               # list of specific genes for the row annotation
row_anno_number = 4                 # number of automatic row annotations per cluster
fontsize_title = 15                 # fontsize the heatmap title
fontsize_rowAnnotation = 8          # row annotation
fontsize_columnNames = 8            # column names
fontsize_rowNames = 4               # row names,
fontsize_cluster_labels = 8         # cluster labels
fontsize_group_annotation = 9       # group annotation title
fontsize_group_annotation_legend = 9 # group annotation legend title
fontsize_group_annotation_labels = 8 # annotation legend labels
fontsize_heatmap_legend = 9         # heatmap legend
fontsize_heatmap_legend_labels = 8 # heatmap legend labels
title_heatmapLegend = "Expression" # changeable title of the legend, default "Expression"
WidthNum = 6                        # heatmap width
HeightNum = 8                       # heatmap height
UnitSize = "cm"                     # heatmap unit for sizes

hm <- adv_Heatmap(ncounts_matrix, seed = seed, column_name = column_name, colorPalette = colorPalette,
                  cluster_method = cluster_method, cluster_rows = cluster_rows,
                  cluster_columns = cluster_columns, k_row = k_row, k_col = k_col,
                  sample_metadata = sample_metadata, annotation_colors = annotation_colors,
                  annotation_name_side = annotation_name_side, show_row_names = show_row_names,
                  show_column_names = show_column_names, row_annotation = row_annotation,
                  row_annotation_method = row_annotation_method, row_anno_names = row_anno_names,
                  row_anno_number = row_anno_number, fontsize_title = fontsize_title,
                  fontsize_rowAnnotation = fontsize_rowAnnotation,
                  fontsize_columnNames = fontsize_columnNames, fontsize_rowNames = fontsize_rowNames,
                  fontsize_cluster_labels = fontsize_cluster_labels,
                  fontsize_group_annotation = fontsize_group_annotation,
                  fontsize_group_annotation_legend = fontsize_group_annotation_legend,
                  fontsize_group_annotation_labels = fontsize_group_annotation_labels,
                  fontsize_heatmap_legend = fontsize_heatmap_legend,
                  fontsize_heatmap_legend_labels = fontsize_heatmap_legend_labels,
                  title_heatmapLegend = title_heatmapLegend, WidthNum = WidthNum,
                  HeightNum = HeightNum, UnitSize = UnitSize)
```

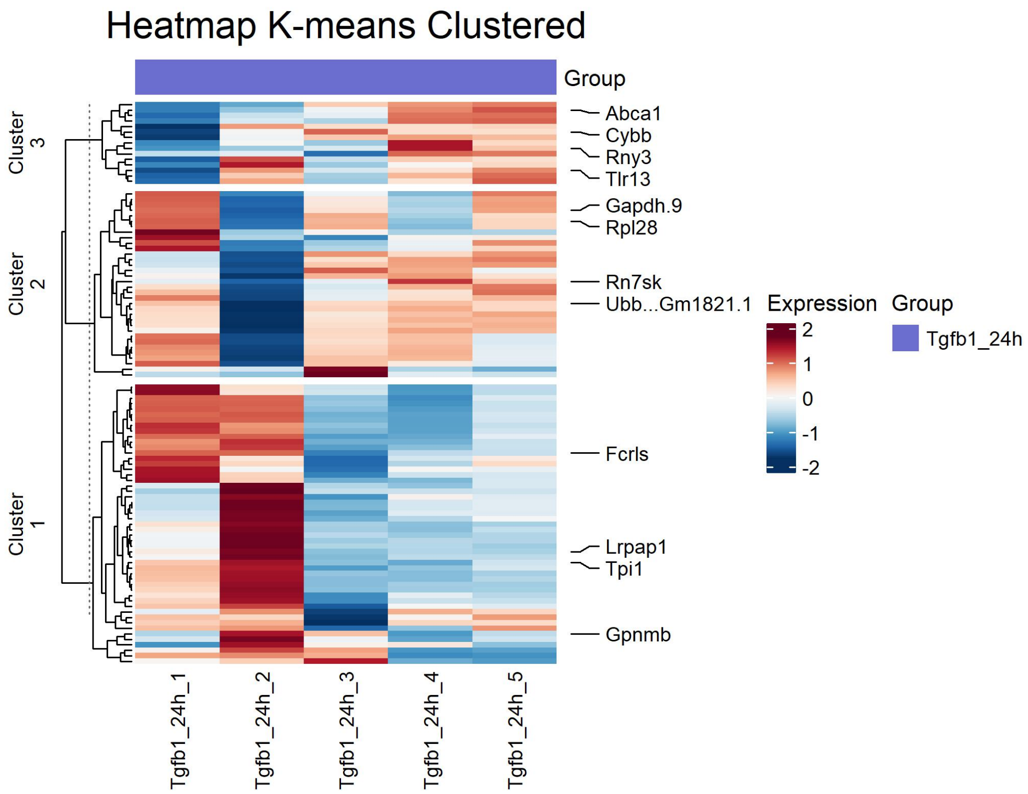

- an advanced heatmap can be generated based on hierarchical clustering

```
# parameters and their options in adv_Heatmap()
ncounts_matrix <- scaled_all_counts  # input matrix
seed <- 1                            # sets seed
column_name <- "Heatmap Hierarchically Clustered"  # name for heatmap
colorPalette <- "RdBu"               # available color palettes from RColorBrewer ()
cluster_method <- "hierarchical"     # cluster methods, either "hierarchical" or "kmeans"
```

```r
distance_method <- "euclidean"      # distance matrix method
cluster_rows <- TRUE                # clustering of rows
cluster_columns <- TRUE             # clustering of columns
k_row = NULL                        # splitting of rows in heatmaps using k-means
k_col = NULL                        # splitting of columns in heatmaps using k-means
sample_metadata <- newMetaData      # metadata information
annotation_colors <- group_colors   # list containing group annotation info
annotation_name_side = "right"      # side of annotation name
show_row_names <- FALSE             # show of rownames
show_column_names = TRUE            # show of column names
row_annotation = TRUE               # row annotation
row_annotation_method = "auto"      # set row_annotation method
row_anno_names = NULL               # list of specific genes for the row annotation
row_anno_number = 10                # number of automatic row annotations per cluster
fontsize_title = 15                 # fontsize the heatmap title
fontsize_rowAnnotation = 8          # fontsize of the optional row annotation
fontsize_columnNames = 8            # fontsize of the optional column names
fontsize_rowNames = 4               # fontsize of the optional row names
fontsize_cluster_labels = 8         # fontsize of the cluster labels
fontsize_group_annotation = 9       # font size of the group annotation title
fontsize_group_annotation_legend = 9 # fontsize of optional group annotation legend title
fontsize_group_annotation_labels = 8 # fontsize of optional group annotation legend labels
fontsize_heatmap_legend = 9         # fontsize of heatmap legend
fontsize_heatmap_legend_labels = 8 # fontsize of heatmap legend labels
title_heatmapLegend = "Expression" # title of the legend
WidthNum = 6                        # heatmap width
HeightNum = 8                       # heatmap height
UnitSize = "cm"                     # heatmap unit for sizes

hm <- adv_Heatmap(ncounts_matrix, seed = seed, column_name = column_name, colorPalette = colorPalette,
                  cluster_method = cluster_method, cluster_rows = cluster_rows,
                  cluster_columns = cluster_columns, k_row = k_row, k_col = k_col,
                  sample_metadata = sample_metadata, annotation_colors = annotation_colors,
                  annotation_name_side = annotation_name_side, show_row_names = show_row_names,
                  show_column_names = show_column_names, row_annotation = row_annotation,
                  row_annotation_method = row_annotation_method, row_anno_names = row_anno_names,
                  row_anno_number = row_anno_number, fontsize_title = fontsize_title,
                  fontsize_rowAnnotation = fontsize_rowAnnotation,
                  fontsize_columnNames = fontsize_columnNames, fontsize_rowNames = fontsize_rowNames,
                  fontsize_cluster_labels = fontsize_cluster_labels,
                  fontsize_group_annotation = fontsize_group_annotation,
                  fontsize_group_annotation_legend = fontsize_group_annotation_legend,
                  fontsize_group_annotation_labels = fontsize_group_annotation_labels,
                  fontsize_heatmap_legend = fontsize_heatmap_legend,
                  fontsize_heatmap_legend_labels = fontsize_heatmap_legend_labels,
                  title_heatmapLegend = title_heatmapLegend, WidthNum = WidthNum,
                  HeightNum = HeightNum, UnitSize = UnitSize)
```

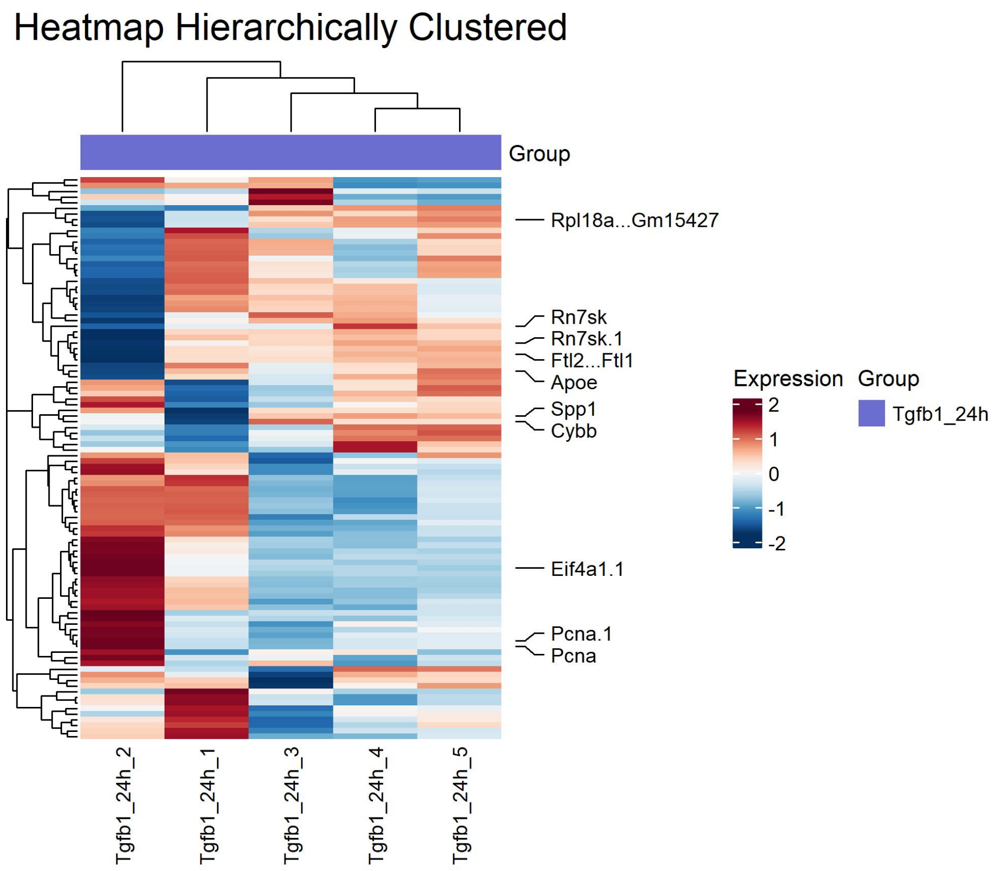
Heatmap Hierarchically Clustered
Group
Rpl18a...Gm15427
Rn7sk
Rn7sk.1
Ftl2...Ftl1
Apoe
Spp1
Cybb
Eif4a1.1
Pcna.1
Pcna
Tgfb1_24h_2
Tgfb1_24h_1
Tgfb1_24h_3
Tgfb1_24h_4
Tgfb1_24h_5
Expression
2
1
0
-1
-2
Group
Tgfb1_24h
